# The Necessity of Prenatal Diagnosis by CMA for the Women with NIPS-Positive Results

**DOI:** 10.1155/2020/2145701

**Published:** 2020-08-29

**Authors:** Jun Xu, Ying Xue, Jing Wang, Qin Zhou, Bin Zhang, Bin Yu, Ting Wang

**Affiliations:** ^1^Changzhou Maternity and Child Health Care Hospital Affiliated to Nanjing Medical University, No. 16 Ding Xiang Road, Changzhou, 213003 Jiangsu Province, China; ^2^The Affiliated Suzhou Hospital of Nanjing Medical University, No. 26 Dao Qian Road, Suzhou, 215000 Jiangsu Province, China

## Abstract

**Objective:**

To retrospectively analyze the results of prenatal diagnoses of noninvasive prenatal screening- (NIPS) positive pregnant women and discuss whether there is a need for chromosomal microarray analysis (CMA).

**Methods:**

The study recruited 1,019 NIPS-positive women from two prenatal diagnostic centers. Based on clinical advice, they opted for traditional karyotype analysis or CMA. Single nucleotide polymorphism array testing was performed on a commercial 750K microarray chip (Affymetrix CytoScan 750K Array).

**Results:**

Of the NIPS-positive women, 761 (74.7%) accepted the prenatal diagnosis. There were 418 (54.9%) abnormal results, and most (99.5%) were chromosome aneuploidy or structural abnormalities. Only three cases were confirmed as pathogenic copy number variation (CNVs), which were found only with CMA and not by karyotype analysis. Fifteen women were variants of uncertain significance (VUS) CNV. In addition, 300 women selected opted for both karyotype analysis and CMA for prenatal diagnosis: in 275 (91.7%) cases, the results of the two modalities were consistent, while in the remaining 25, they were not. In three cases, the additional positive results obtained with CMA were potentially clinically significant.

**Conclusions:**

CMA may not be useful for many women positive for trisomy 21/18/13 based on NIPS results, because traditional karyotype analysis can identify most problems. However, it can yield important additional findings in women positive for fetal sex chromosome aneuploidy (SCA). Further clinical studies are needed to confirm these findings.

## 1. Introduction

Noninvasive prenatal screening (NIPS) is widely used to screen for trisomies 21 (T21), 18 (T18), and 13 (T13) and shows high accuracy and a low rate of false positives [1–4]. NIPS is also playing an increasingly important role in prenatal screening for other fetal genetic diseases, including fetal sex chromosome aneuploidy (SCA) [5], fetal microdeletions/microduplications [6, 7], and monogenic inherited diseases [8]. The clinical strategies for NIPS differ slightly among countries according to the available technology, economic conditions, education level of the population, legislation, and so on. NIPS is already widely used as a first-line screening test in some countries. In China, however, it is still considered a second-line prenatal screening test. NIPS is mainly used to screen women determined as being at intermediate risk based on serological screening, or as an alternative screening test for women at high risk, or of advanced maternal age, who reject invasive prenatal diagnostic tests. As both a first- and second-line screening test, NIPS is becoming an increasingly important and effective prenatal screening approach for fetal genetic diseases.

Due to technological advances, pregnant women are increasingly willing to undergo NIPS, although it is also clear that an interventional prenatal diagnosis is required after NIPS-positive results [9]. However, there has been scant research on the best methods for prenatal diagnosis after NIPS. Given the increasing clinical application of NIPS, this issue will become more important. In general, the choice of diagnostic testing depends on the size of the anomaly or degree of structural rearrangement. Current methods include chromosome analysis, chromosomal microarray analysis (CMA), fluorescence *in situ* hybridization (FISH), and rapid polymerase chain reaction (PCR) [10]. Standard karyotype analysis is the traditional prenatal diagnostic method which can detect major chromosomal abnormalities, such as aneuploidy, unbalanced rearrangements, Robertsonian translocation, and mosaicism. Recently, CMA, a high-resolution genomic technology, has also been applied for prenatal diagnosis of genetic disorders. CMA has considerable diagnostic and prognostic value but has not yet fully replaced karyotype analysis. It confers diagnostic benefits by revealing submicroscopic imbalances or copy number variation (CNV), which cannot be detected by standard karyotype analysis [11, 12]. There are marked differences in the prenatal diagnostic follow-up of NIPS among countries, and different modalities are used depending on the NIPS results obtained. Currently, karyotype analysis and CMA are the most common methods for prenatal diagnosis in China. However, no study has examined the preferred prenatal diagnostic approach for NIPS-positive pregnant women in China.

As a preliminary discussion of this interesting question, this study examined the clinical data of 761 NIPS-positive women who accepted the prenatal diagnosis and underwent traditional karyotype analysis or CMA testing to generate clinical data to guide the choice of prenatal diagnostic methods.

## 2. Materials and Methods

### 2.1. Patients and Design

The study design and protocol were reviewed and approved by the ethics committee of Changzhou Maternity and Child Health Care Hospital affiliated to Nanjing Medical University (No. 2017003). All pregnant women received genetic counseling and provided informed consent before testing.

From February 2015 to December 2018, 68,188 pregnant women underwent NIPS.

The technical procedures for NIPS were reported previously [1]. 1,019 cases got the NIPS-positive results. We recruited the women with positive results to this study. The participants were recruited from two prenatal diagnostic centers: Changzhou Maternity and Child Health Care Hospital affiliated to Nanjing Medical University and The Affiliated Suzhou Hospital of Nanjing Medical University. The pregnant women were aged 25–37 years old and were between 14 (and 1 day) and 22 (and 1 day) gestational weeks. They were promptly recalled for genetic counseling, and most of them accepted the prenatal diagnosis. Based on clinical advice, they agreed to traditional karyotype analysis or CMA for further testing. Both centers used the same detection platform, experimental scheme, and quality control standards. They all participated in the laboratory quality control evaluation plan. We compared the characteristics of NIPS, CMA, and conventional karyotyping in Table 1.

### 2.2. Karyotype Analysis for Prenatal Diagnosis

The pregnant women underwent amniocentesis at a suitable gestational stage (18–23 weeks) in the prenatal diagnostic centers. The cytogenetic prenatal diagnostic methods used for karyotype analysis were similar to those applied in our previous report [1, 9]. All tests were performed independently by two individuals using two cell culture systems: CHANG Amnio (Emergo Europe, The Hague, The Netherlands) and amniotic fluid cell culture medium (Hangzhou Baorong Science and Technology, Hangzhou, China). After cell culture and sample preparation, a GSL-120 instrument (Leica Biosystems, Richmond, IL, USA) and CytoVision software (Leica) were used for chromosome karyotype scanning and analysis. At least five cell karyotypes were analyzed, and 20 karyotypes were counted. For the cases with chromosome mosaicism, 60 to 100 karyotypes were counted.

### 2.3. Chromosomal Microarray Analysis for Prenatal Diagnosis

The prenatal CMA procedure was as described in our previous study [13]. After amniotic fluid was collected, DNA was extracted using a QIAamp DNA Mini Kit (Qiagen, Valencia, CA, USA). Then, 250 ng DNA was amplified, labeled, and hybridized to the GCS 3000Dx v.2 platform (Affymetrix, Santa Clara, CA, USA). The SNP array test was processed with a commercial 750K microarray chip (CytoScan 750K Array; Affymetrix). After hybridization with fragmented DNA, the chip was washed with buffer and scanned with a laser scanner. The data were analyzed using Chromosome Analysis Suite v3.2 (Affymetrix).

### 2.4. Statistical Analysis

The data were analyzed using Empower Stats (X&Y Solutions, Boston, MA, USA) and R (http://www.R-project.org) [14]. Age and gestational age are expressed as the median, 2.5^th^ percentile, and 97.5^th^ percentile. *P* < 0.05 indicated statistical significance.

## 3. Results

**Figure 1:**
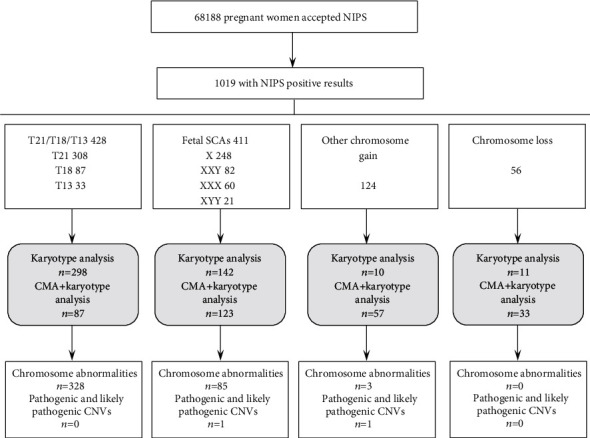
The general results of the present study.

In this study, 68,188 pregnant women underwent NIPS at two prenatal diagnostic centers and 1,019 got positive results. According to the NIPS results, they were divided into four groups: T21/T18/T13 aneuploidy (428 cases), fetal SCA abnormalities (411 cases), gain of another chromosome (124 cases), and chromosome loss (excluding X and Y; 56 cases). The women with positive results were recalled and consulted by clinicians. In all, 761 (74.7%) women accepted the prenatal diagnosis. Of the women with T21/T18/T13-positive results, 385 (90.0%) accepted the prenatal diagnosis. Another 18 cases showed severe fetal ultrasound abnormalities or miscarried. In the other groups, the rates of prenatal diagnosis were lower, ranging from 54.0% to 78.6%. Of the 761 pregnant women, 461 requested only traditional karyotype analysis, while 300 also underwent additional CMA. The acceptance rate of CMA was lowest in the women with T21/T18/T13-positive results, at 22.6% (87/385). The other pregnant women were more willing to undergo CMA, including 46.4% in the group with fetal SCA abnormalities, 75% in the chromosome loss group, and 85.1% in the group that gained another chromosome.  Figure 1 summarizes the study results.

The prenatal diagnostic tests revealed 418 (54.9%) abnormal results (Table 2 and Supplementary Table (available here). Most (416, 99.5%) of these cases showed chromosome aneuploidy or structural abnormalities, which could be detected by both traditional karyotype analysis and CMA. Only three cases were confirmed as pathogenic CNVs, as detected by CMA but not by karyotype analysis. One case was confirmed as arr[hg19] Xp21.3p11.4 (27,089,486-38,583,792)x2. The NIPS result was Chr:X+Y, and the *Z*-values were 0.57 for the X chromosome and 53.49 for the Y chromosome. There was an 11.5 Mb duplication (nt. 27,089,486-38,583,792) in the Xp21.3p11.4 region, including 21 OMIM genes. To investigate genotype-phenotype correlations, we obtained additional cases with overlapping Xp21.3p11.4 duplication via a systematic literature review. Congenital malformations were seen, varying according to the size and position of the duplication; in one case, intellectual deficiency (ID) was seen but, after genetic counseling, the woman insisted on continuing the pregnancy and gave birth to a boy. After 15 months, the child has shown no signs of intellectual disability or other manifestations. Another case was confirmed as arr[hg19] 16p13.11p12.3 (15,319,277-18,242,713)x3. The NIPS result was of chr16+, and the *Z*-value for chr16 was 2.08. There was a 2.923 Mb duplication (nt. 15,319,277-18,242,713) in the 16p13.11p12.3 region, including seven OMIM genes. The duplication contained region 16p13.11, as seen in recurrent microduplication syndrome, which is genetically heterogeneous and has a low incidence, mainly manifesting as physical retardation and linguistic and learning disabilities. Some patients have no, or mild, clinical phenotypes and parents with no obvious genetic abnormalities. The third case was confirmed as arr[hg19] Xp22.31 (6,455,151-8,143,509)x3. The child has not yet shown growth retardation or other manifestations after 18 months of follow-up.

We also found that 15 cases were variants of uncertain significance (VUS) CNV. These cases have been followed for between 5 months and 2 years. One mother terminated the pregnancy due to severe fetal growth retardation. Among the 17 cases whose babies were born, one infant had neonatal intestinal obstruction. The others have shown no abnormalities during their growth and development (Table 3).

In this study, 300 women selected both karyotype analysis and CMA for prenatal diagnosis (Table 4). The results were consistent in 275 cases (91.7%), including 179 negative cases and 96 positive cases, while 25 women had inconsistent results. Eighteen cases had additional positive CMA results, but most were LB or VUS. Only four cases were potentially clinically significant. In addition to three cases with pathogenic CNVs (as mentioned above), one case was confirmed as chr8 uniparental disomy (UPD). Her result suggested that the signal of chromosome 8 increased, and she chose to continue her pregnancy, and there were no complications or ultrasound abnormalities during the pregnancy. We have followed the infant for 16 months, and no abnormalities have been found. Seven women showed additional positive results in the traditional karyotype analysis. Most cases showed chromosome polymorphism, such as qh+ or pstk+. Two cases showed low-ratio chromosome mosaics (one was 45,X[4]/46,XX[46], and the other was 46,XX[48]/47,XXX[2]. The mosaicism rates were 8% and 4%, respectively). There was one case of 46,XX,inv(7)(q33q35). No defects would be expected in these cases, but long-term follow-up is needed in case significant clinical pathogenicity develops.

## 4. Discussion

Regarding the diagnostic approach that NIPS-positive pregnant women should choose after a prenatal diagnosis, the American College of Medical Genetics and Genomics Clinical Laboratory Practice Resources state that different follow-up diagnostic testing should be provided depending on the NIPS results. For example, chromosome analysis and CMA are recommended for T21/T18/T13 aneuploidies. CMA is recommended for small copy number changes [10]. In China, karyotype analysis was long considered the first choice for prenatal diagnosis. In recent years, however, an increasing number of pregnant women have also opted for CMA at the same time, especially those with an abnormal prenatal ultrasound. However, no study has focused on the diagnostic testing conducted after NIPS-positive results.

CMA is being increasingly applied for prenatal diagnosis and is thought to have better diagnostic efficacy than traditional karyotype analysis because it can detect submicroscopic imbalances or CNV. According to recent reports, CMA increases the prenatal diagnostic rate by 1~6% [11, 15–17]. However, CMA has not fully replaced traditional karyotyping because it also has some limitations [13]. For example, it cannot detect balanced structural rearrangements. CMA also requires advanced equipment and is more expensive, which serve as barriers in genetic counseling. In this study, we firstly discussed whether CMA was necessary for prenatal diagnosis in NIPS-positive pregnant women. Although pregnant women are increasingly willing to undergo prenatal CMA, this has not resulted in a meaningful increase in the rate of discovery of NIPS-positive women. Of the 418 cases with pathogenic abnormalities in this study, only three cases were confirmed as pathogenic or likely pathogenic CNVs, where this was revealed only by CMA and not by karyotype analysis. The CMA detected only three additional meaningful results, which accounted for 0.71% of all anomalies. CMA also revealed some CNVs. Although such findings pose difficulties in the context of prenatal genetic counseling, they are nevertheless valuable to the human genetic database.

Our results can be summarized as follows. First, for NIPS-positive women with T21/T18/T13, CMA may not be particularly valuable, because traditional karyotype analysis can identify most problems. NIPS is very effective in screening for T21/T18/T13 aneuploidy. In this study, 385 pregnant women received a prenatal diagnosis, and the results were essentially consistent with NIPS. The respective detection and false-positive rates were, respectively, 99.7% and 0.04% for T21, 97.9% and 0.04% for T18, and 99.0% and 0.04% for T13 [4, 18]. The application of CMA did not result in additional discoveries, such as microdeletion or microduplication. However, we cannot conclude that CMA is unnecessary, because only a limited number of women requested it; this preliminary conclusion requires clinical confirmation. Second, for women with NIPS-positive results for fetal SCA, CMA may provide some additional findings, but further research is needed to confirm this. NIPS are important for prenatal diagnosis of sex chromosome abnormalities. However, there are many related ethical problems. Previously, we found that the overall positive predictive value (PPV) of NIPS for SCA was 54.5%, but there were significant differences among SCA diseases [5]. In the current study, CMA detected an additional case of pathogenic CNV. Third, the diagnostic accuracy of NIPS was poor for conditions such as autosomal aneuploidies and chromosome loss. Technical improvements are required for NIPS. Liang et al. optimized molecular techniques by modifying key steps in the original NIPS process and reported that NIPS-Plus yielded high PPVs for common aneuploidies and DiGeorge syndrome and moderate PPVs for other microdeletion/microduplication syndromes [7]. Fourth, NIPS-positive pregnant women might benefit from either karyotype analysis or CMA alone as the prenatal diagnostic method. Our results indicated that both modalities can yield important additional findings, unless the NIPS result strongly suggests a high risk of T21/T18/T13. Fifth, prenatal CMA can shorten the reporting time and ease the burden on karyotype analysis, where amniotic fluid culture occasionally fails.

In conclusion, it is important to determine whether NIPS-positive pregnant women need CMA for prenatal diagnosis. We believe that the prenatal diagnostic approach used for NIPS-positive pregnant women should vary on a case-by-case basis. CMA may not be useful for women who are NIPS-positive for T21/T18/T13, whereas it may yield important additional findings in women positive for fetal SCA. Further clinical studies are needed to confirm these findings.

## Figures and Tables

**Table 1:** The comparison between NIPS, CMA, and conventional karyotyping.

	NIPS	Karyotype analysis	CMA
Technology positioning	Prenatal screening	Prenatal diagnosis	Prenatal diagnosis
Cell culture	N	Y	N
Cost	Middle	Low	High
Reporting time	1~2 weeks	4~5 weeks	1~2 weeks
Aneuploid	Y (mainly to T21/T18/T13)	Y	Y
Large fragment (>10 M)	Sometime	Y	Y
Microdeletion/microduplication (<10 M)	Sometime	N	Y
Balanced translocation	N	Y	N
Unbalanced translocation	N	Y	Y
Loss of heterozygosity (LOH)	N	N	Y
Uniparental disomy (UPD)	N	N	Y

This table only compared the application of these three techniques in prenatal screening and diagnosis.

**Table 2:** The results of prenatal diagnosis in the pregnant women with NIPS-positive results.

Group	*N*	Prenatal diagnosis	Chromosome abnormalities		CNVs					Polymorphism
			Aneuploidy	Structural abnormalities	P	LP	VUS	LB	B	
NIPS reported as T21/T18/T13 positive	428	385	305	23	0	0	0	0	0	0
NIPS reported as SCA positive	411	265	71	13	1	0	6	0	0	4
NIPS reported as other chromosome aneuploidy positive	124	67	0	3	2	0	5	3	0	0
NIPS reported as chromosome signal reduction	56	44	0	0	0	0	4	0	0	1

CNVs: copy number variations; P: pathogenic; LP: likely pathogenic; VUS: variants of uncertain significance; LB: likely benign; B: benign. The detailed results are shown in Supplementary table 1.

**Table 3:** The results of CNVs in the pregnant women with NIPS-positive results.

Case	Age	NIPS				Amniotic fluid			Pregnant outcomes
		Gestational weeks	cffDNA%	*Z*	Results	Gestational weeks	Results	CNVs	
1	27	18 + 1	—	X: 0.57 Y: 53.49	ChrX+(Y)	20 + 2	arr[hg19] Xp21.3p11.4 (27,089,486-38,583,792)x2	P	Following up^a^
2	28	16 + 2	13.10	2.08	Chr16+	21 + 2	arr[hg19] 16p13.11p12.3 (15,319,277-18,242,713)x3	P	Following up^b^
3	30	17 + 5	29.18	7.25	Chr13+	18 + 4	arr[hg19] Xp22.31 (6,455,151-8,143,509)x3	P	Following up^c^
4	36	17 + 6	9.74	X: -3.99 Y: 48.81	ChrX-Y	18 + 6	arr[hg19] Xp11.4 (41,368,239-41,630,429)x2	VUS	Terminal pregnancy^d^
5	31	17 + 6	11.35	X: -3.72 Y: -0.56	ChrX-	19 + 5	arr[hg19] 7q21.3 (96,697,458-97,803,931)x3	VUS	Normal
6	34	19 + 4	16.57	X: -54.68 Y: 0.32	ChrX-	19 + 6	arr[hg19] 16p12.2 (21,405,327-21,816,543)x1 arr[hg19] 17q12q21.32 (33,878,223-46,597,013) hmz	VUS	Normal
7	36	18 + 2	7.2	X: -3.22 Y: 0.21	ChrX-	21 + 4	arr[hg19] 15q14 (34,252,925-36,783,190)x3	VUS	Normal
8	32	18 + 3	—	6.33	Chr9+	21 + 6	arr[hg19] 9q31.1q33.1 (107,923,508-121,624,320)x3	VUS	Neonatal intestinal obstruction
9	24	15 + 5	14.84	4.32	Chr21+	18 + 4	arr[hg19] 21q21.2 (24,247,587-26,223,391)x3	VUS	Normal
10	28	14 + 1	5.21	-5.14	Chr21-	28 + 0	arr[hg19] 5q14.3q15 (83,979,073-95,066,296) hmz	VUS	Normal
11	32	17 + 3	—	-5.76	Chr13-	22 + 3	arr[hg19] 2p25.3 (314,374-850,139)x3	VUS	Normal
12	27	19 + 4	15.76	-3.10	Chr9-	22 + 2	arr[hg19] 9p21.1 (28,742,800-29,780,373)x1 arr[hg19] 9p21.1 (30,547,485-31,996,569)x1	VUS	Normal
13	22	20 + 0	7.73	-3.28	Chr12-	25 + 6	arr[hg19] 12q14.1 (58,458,371-61,582,295)x1	VUS	Normal
14	30	19 + 5	14.05	4.28	Chr3+	22 + 1	arr[hg19] Yq11.221q11.23 (19,563,599-26,273,936)x2	VUS	Normal
15	25	17 + 0	7.88	0.79	Chr2+	20 + 5	arr[hg19] 2p12 (78,631,709-79,973,436)x3	VUS	Normal
16	24	16 + 6	17.52	4.51	Chr12+	20 + 4	arr[hg19] 12q21.2q21.31 (78,770,625-84,470,319)x3	VUS	Normal
17	37	16 + 2	13.11	1.21	Chr7+	21 + 1	arr[hg19] 7q31.31 (117,614,219-118,512,894)x3	LB	Normal
18	31	16 + 6	7.65	0.56	Chr6+	19 + 0	arr[hg19] 6q26q27 (164,292,513-165,017,873)x3	LB	Normal
19	39	22 + 1	17.61	6.09	Chr11+	24 + 5	arr[hg19] 11p11.12 (49,193,984-51,238,712)x3	LB	Normal
20	26	14 + 2	11.26	3.35	ChrX+(Y)	20 + 2	arr[hg19] Xq21.2q21.31 (85,393,530-89,136,096)x2 arr[hg19] Xq26.3q27.1 (137,899,736-139,867,922)x2 arr[hg19] Xq27.3 (142,856,383-144,729,2480)x2	VUS	Normal
21	26	17 + 0	21.11	-7.77	ChrX-	19 + 5	arr[hg19] 8q23.3 (113,624,542-115,587,168)x3	VUS	Normal

CNVs: copy number variations; P: pathogenic; LP: likely pathogenic; VUS: variants of uncertain significance; LB: likely benign; B: benign. ^a^The women insisted on continuing pregnancy, and a boy was born. After 15 months of follow-up, the child has not yet shown intellectual disability and other manifestations. ^b^After 18 months of follow-up, the child has not yet shown growth retardation or other manifestations. ^c^After 12 months of follow-up, the child has not yet shown growth retardation or other manifestations. ^d^Due to severe fetal growth retardation, the mother decided to terminate the pregnancy. Normal: the pregnant woman decided to continue the pregnancy, and the babies were born. After 5 months to 2 years of follow-up, there was no obvious abnormality in the growth and development of children.

**Table 4:** The results of prenatal diagnosis in the women who selected both karyotype analysis and CMA.

Group	Number	Chromosome abnormalities	CNVs
K+C+	96	96	3
K-C+	18	1^a^	17^b^
K+C-	7	7^c^	0
K-C-	179	0	0
Total	300	104	21

K+C+: both results of karyotype analysis and CMA were positive; K-C+: the results of CMA were positive, while karyotype analysis results were negative; K+C-: the results were karyotype analysis positive, while CMA results were negative; K-C-: both results of karyotype analysis and CMA were negative. ^a^One case was chr8 uniparental disomy (UPD). ^b^Two cases were pathogenic or likely pathogenic CNVs. Three cases were likely benign CNVs. Others were VUS. ^c^Two cases were low-ratio chromosome mosaics. One case was 46,XX,inv(7)(q33q35). Four cases were chromosome polymorphism.

## Data Availability

The data used to support the findings of this study are available from the corresponding author upon request.

## Supplementary Materials

Supplementary Materials Supplementary table: the abnormal results of prenatal diagnosis.
