# Supplementary material for: The Necessity of Prenatal Diagnosis by CMA for the Women with NIPS-Positive Results
Source: Int J Genomics. 2020 Aug 29;2020:2145701. doi: 10.1155/2020/2145701 (PMC7481948; doi:10.1155/2020/2145701)
Supplement: Supplementary Materials — Supplementary table: the abnormal results of prenatal diagnosis. [file 2145701.f1.doc]

**Supplementary table. The abnormal results of prenatal diagnosis**

| **Group** | **Chromosome abnormalities** | | **CNVs** | | | | **Polymorphism** |
| --- | --- | --- | --- | --- | --- | --- | --- |
|  | **number abnormalities** | **structural abnormalities** | **P** | **LP** | **VUS** | **LB** |  |
| NIPS reported as T21/T18/T13 positive | T21 242  T18 56  T13 7 | 46,XX,rob(21;21)  46,XY,rob(21;21)  46, XY,add(9)(p24)  46, XY,rob(14;21)  45, XX,der(5;13)  45,XY,-21[11]/46,XY,r(21)[39]  46,XX,dic(Y;13),+21  46,XX,+21,der(21;21)(q10;q10)  46,XY,+21,rob(21;21)(q10;q10)  46,XY,+21,rob(21;21)(q10;q10)  46,XX,+21,rob(21;32)(q10;q10)  46,XX,+der(21;21)(p11;q11)  46,XY,-21,+rob(21;21)(q10;q10)  46,XX, rob(13;21),+21  46,XY,rob(14;21)(q10;q10),+21  47,XX,+21[26]/47,XX,t(18;20)(q23;q12),+21[4]  47,XY,+21[28]/46,XY[22]*2  47,XX,inv(9)(p12q13),+21  47,XY, t(2;3)(q33;q27),+21  46,XX,dup(18)(p11q22)  47,XY,+18[2]/46,XY[68]  46,XX,der(13;22)(q10;q10),+13 | 0 | 0 | 0 | 0 | 0 |
| NIPS reported as SCAs positive | X 13  XXY 28  XXX 21  XYY 9 | 45,X[43]/46,X,+mar[7]  45,X[2]/46,XX[18]  45,X[30]/46,XX[20]  45,X[38]/47,XXX[12]  45,X[4]/46,XX[46]  45,X[9]/46,XX[21]  45X[11]/46,x,+mar[43]  46,XX,inv(7)(q33q35)  46,XX,inv(9)(p12;q13)  46,XX/47,XXX mosaic  47,XXX[12]/45,X[8]  47,XXX[9]/45,X[41]  46,X,der（X;?Y）（p22.2；q11.1） | arr[hg19] Xp21.3p11.4(27,089,486-38,583,792)x2 | 0 | arr[hg19]Xp11.4(41,368,239-41,630,429)x2  arr[hg19]7q21.3(96,697,458-97,803,931)x3  arr[hg19]16p12.2(21,405,327-21,816,543)x1，arr[hg19] 17q12q21.32(33,878,223-46,597,013) hmz  arr[hg19]15q14(34,252,925-36,783,190)x3  arr[hg19]8q23.3(113.624.542-115.587.168)x3  arr[hg19]Xq21.2q21.31(85,393,530-89,136,096)x2,arr[hg19]Xq26.3q27.1(137,899,736-139,867,922)x2;arr[hg19]Xq27.3(142,856,383-144,729,2480x2 | 0 | 46,XX,9qh+  46,XX,19h+  46,XY,Yqh+  46,XX,22pstk+ |
| NIPS reported as other chromosome aneuploidy  positive | 0 | 46,XY,add9(q33)  46,XX,t(1;6)(p22;p12)  Chr8 UPD | arr[hg19]Xp22.31(6,455,151-8,143,509)x3  arr[hg19]16p13.11p12.3(15,319,277-18,242,713)*3 |  | arr[hg19]9q31.1q33.1(107,923,508-121,624,320)x3  arr[hg19]21q21.2(24,247,587-26,223,391)x3  arr[hg19]Yq11.221q11.23(19,563,599-26,273,936) x 2  arr[hg19]2p12(78,631,709-79,973,436) x 3  arr[hg19]12q21.2q21.31（78,770,625-84,470,319）x 3 | arr[hg19]7q31.31(117,614,219-118,512,894) x 3  arr[hg19]6q26q27(164,292,513-165,017,873) x 3  arr[hg19]11p11.12(49,193,984-51,238,712)x3 | 0 |
| NIPS reported as chromosome signal reduction | 0 | 0 | 0 | 0 | arr[hg19]2p25.3(314,374-850,139)x3  arr[hg19]5q14.3q15(83,979,073-95,066,296) hmz  arr[hg19]9p21.1(28,742,800-29,780,373)x1，arr[hg19] 9p21.1(30,547,485-31,996,569)x1  arr[hg19]12q14.1（58,458,371-61,582,295）x 1 | 0 | 46,XX,16qh+ |
